# Retro-Mode Scanning Laser Ophthalmoscopy Planning for Navigated Macular Laser Photocoagulation in Macular Edema

**DOI:** 10.1155/2016/3726353

**Published:** 2016-02-17

**Authors:** Ernest V. Boiko, Dmitrii S. Maltsev

**Affiliations:** ^1^St. Petersburg Branch of the S. Fyodorov Eye Microsurgery Federal State Institution, 21 Yaroslav Gashek Street, Saint Petersburg 192283, Russia; ^2^Department of Ophthalmology, Military Medical Academy, 5 Klinicheskaya Street, Saint Petersburg 194044, Russia

## Abstract

*Purpose*. To compare treatment areas and navigated macular laser photocoagulation (MLP) plans suggested by retro-mode scanning laser ophthalmoscopy (RM-SLO) image versus optical coherence tomography (OCT) central retinal thickness map and treatment planning among retina specialists.* Methods*. Thirty-nine eyes with diabetic or branch retinal vein occlusion-related ME undergoing navigated MLP with navigated photocoagulator had OCT and RM-SLO taken. OCT map and RM-SLO image were imported to the photocoagulator and aligned onto the retina. Two retina specialists placed laser spot marks separately based on OCT and RM-SLO images in a random fashion. The spots placed by each physician were compared between OCT and RM-SLO and among physicians. The areas of retinal edema on OCT and RM-SLO of the same eye were also compared.* Results*. The average number of laser spots using RM-SLO and OCT template was 189.6 ± 77.4 and 136.6 ± 46.8, respectively, *P* = 0.003. The average area of edema on RM-SLO image was larger than that on OCT map (14.5 ± 3.9 mm^2^ versus 10.3 ± 2.8 mm^2^, *P* = 0.005) because of a larger scanning area. There was narrow variability in treatment planning among retina specialists for both RM-SLO (*P* = 0.13) and OCT (*P* = 0.19).* Conclusion*. The RM-SLO image superimposed onto the fundus of the same eye can be used to guide MLP with narrow variability in treatment planning among retina specialists. The treatment areas suggested by RM-SLO-guided MLP plans for ME were shown to be larger than those suggested by OCT-guided plans.

## 1. Introduction

Laser treatment [1] and intravitreal therapy with antiangiogenic agents and steroids [2] are the two standard treatment options for macular edema (ME) secondary to diabetes and retinal vein occlusion (RVO). The latter option, although shown by multiple studies [3, 4] to be efficacious for this condition, involves numerous intravitreal injections, which is associated with the cumulative risk of endophthalmitis [5]. Additionally, intravitreal antiangiogenic agents and steroids result in an increased financial burden for the patient [6] and increased rates of elevated intraocular pressure and cataract [7], respectively.

Therefore, laser therapy, either alone, as an adjunct to intravitreal antiangiogenic and steroid therapy [1, 2, 8, 9] or as a switching therapy for non-responders to pharmacotherapeutic-only options [10] is still important in treating ME. Central scotoma, loss of central vision, and decreased color vision [11, 12] associated with progressive retinal pigment epithelial atrophy are possible adverse effects of macular laser photocoagulation (MLP). Novel navigated MLP has however been shown to be safe and to improve accuracy in photocoagulation treatments of diabetic retinopathy lesions compared to conventional manual-technique laser treatment [13].

Currently, the standard approach to MLP involves treating individual-leaking microaneurysms (ILMs) and/or diffuse vascular leakage (DVL) [14] regions. For decades, fluorescein retinal angiography (FA) was the only technique to demonstrate reliably ILMs, DVL regions and associated ME. Since retinal edema develops mainly as a result of retinal vascular leakage [15], in some cases of this disease, FA-guided planning for MLP may be changed for OCT-guided planning. Moreover, OCT-guided planning has been already used for navigated MLP [16–18], and it is reasonable to expect an increase in the use of such an approach. OCT-guided planning for MLP (for macular edema) has shown similar results as FA-guided planning, with visualization of the retinal edema regions of similar area and location [16, 19].

Retro-mode (RM) scanning laser ophthalmoscopy (SLO) is another new method for visualizing the retinal edema regions and also may have the potential to be used to guide navigated MLP. In RM-SLO, the scattered light that passes a deviated aperture gives a shadow to the silhouetted cystoid spaces, allowing for clear visualization of intraretinal cystoid spaces in retinal edema [20].

The purpose of the study was (1) to compare treatment areas suggested by RM-SLO versus OCT and (2) to compare navigated MLP plans using RM-SLO images superimposed onto a color fundus photograph versus OCT thickness map superimposed onto a color fundus photograph among retina specialists.

## 2. Materials and Methods

The study was approved by the Ethics Committee of Military Medical Academy and followed the tenets of the Declaration of Helsinki. All patients gave written informed consent for both participation in the study and for MPL. Before treatment, they were explained the cause of the disease, treatment options available to address macular edema, as well as advantages and disadvantages of these options. Patients' decision in favor of having MLP was free, conscious and voluntary (and based mostly on economic reasons).

The inclusion criteria for this prospective, randomized study included OCT (central foveal B scan) evidence of diabetic or RVO-related macular edema or RM-SLO evidence of retinal edema (diabetic or RVO-related macular edema) for which MLP was to be performed. Exclusion criteria included evidence of acute or chronic uveitis, vitreoretinal traction, fibrosis of the internal limiting membrane (with macular involvement), central RVO, and apparent optic media opacity (resulting in OCT images with a Signal Strength Index reduced to less than 40).

### 2.1. MLP Technique

In all eyes of the study, navigated MLP (NAVILAS, OD-OS Inc, Berlin, Germany) with 1% tropicamide (Mydriacyl; Alcon-Couvreur, Puurs, Belgium)-induced mydriasis was planned and performed following RM-SLO and OCT imaging. The MLP-related treatment decisions were to deliver laser to areas of retinal edema, as per Early Treatment Diabetic Retinopathy Study (ETDRS) guidelines. The parameters used included a spot size of 50 *μ*m, burn spacing of 2 burn-widths apart, and areas to avoid including the optic nerve head and central macular 2000-*μ*m diameter area.

### 2.2. RM-SLO Images

RM-SLO image obtained with SLO F-10 (NIDEK, Gamagori, Japan) was imported into the NAVILAS system, superimposed onto the baseline image (i.e., NAVILAS color fundus image), and utilized for treatment planning. In RM-SLO images, retinal edema was defined as the zones with intraretinal microcysts visible as raised structures with clearly defined boundaries.

### 2.3. OCT Maps

An OCT retinal thickness map (Enhanced Macular Map 5 (EMM5) protocol) and background SLO-type fundus photograph (7 mm × 7 mm) were acquired on the spectral domain OCT system (RTVue-100, Optovue, Fremont, CA) and imported into the NAVILAS system to overlay them on a baseline color fundus image and to utilize for treatment planning. In the OCT retinal thickness map, retinal edema was defined as the zones with retinal thickness greater than 250 *μ*m indicated by colors warmer than green. All OCT images were obtained by a single, experienced technician. Macular thickness map artifacts were assessed, focusing on automatic segmentation and off-center errors, and corrected manually as soon as scanning was completed. Errors in automatic segmentation were corrected by manual boundary segmentation of images on B scans. Off-center errors, if any, were corrected through manual repositioning of the foveal center using B scan landmarks that allow its identification. If it was impossible to correct a retinal thickness map manually in a proper way, rescanning and manual post-scan correction of such errors was performed until obtaining an adequate map for the treatment planning for MLP.

### 2.4. Measurement of the Retinal Edema Area and Planning

Two MLP specialists independently measured the area of retinal edema on OCT maps and RM-SLO images in a random order using the ImageJ software (NIH, Bethesda, MD). Additionally, they performed preplanning which involved placing laser spot marks after superimposing the OCT map or RM-SLO image onto the baseline image (i.e., NAVILAS color fundus image). The spots placed by each physician were compared between RM-SLO and OCT and among physicians. In addition, the areas of retinal edema measured by each physician were compared between RM-SLO and OCT and among the physicians.

### 2.5. Statistics

Unless otherwise stated, all the data are expressed as the mean standard deviation (SD). The paired *t*-test was used to compare equivalent parameters (the area of retinal edema and the number of spots placed by the physician) obtained with RM-SLO and OCT. Additionally, it was used to compare the area of retinal edema and the number of spots placed among physicians.

## 3. Results

Thirty-two patients (20 women and 12 men, mean age: 65.3 ± 8.7 years) were included into the study. In 22 patients (29 eyes), the examination revealed diabetic macular edema which had not been treated with MLP. Ten patients (10 eyes) were diagnosed with macular edema secondary to branch RVO.

### 3.1. Quality of OCT Maps and RM-SLO Images

**Figure 1:**
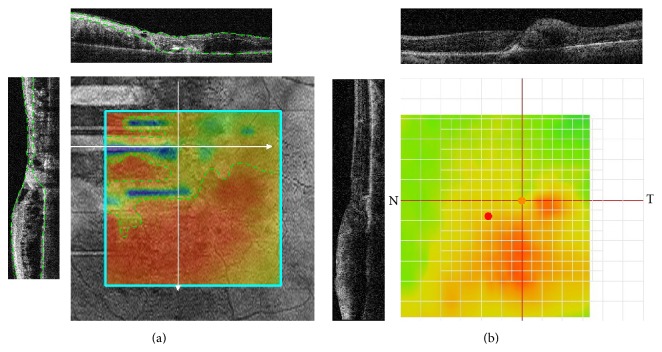
Artifacts in OCT retinal thickness maps that impede OCT-guided planning for navigated MLP. (a) Incorrect automated segmentation (in B scans, a green dashed line represents automated segmentation boundaries; white arrows on the map indicate the locations of B scans) resulting in the occurrence of zones with underestimated retinal thickness (indicated by map colors colder than red and limited by a yellow dashed line). (b) Off-center artifact due to fixation error (green mark represents the true foveal center identified with B scans; red mark represents the foveal center identified automatically; black lines on the map represent the locations of B scans).

**Figure 2:**
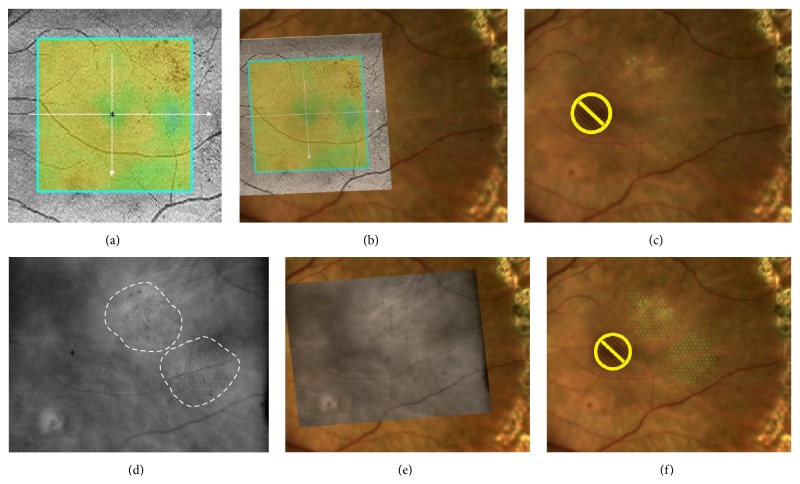
OCT- versus RM-SLO-guided treatment plan for macular laser photocoagulation. (a) Retinal edema is visualized on the OCT retinal thickness map (asterisk represents the foveal center). (b) OCT map is superimposed onto the baseline image. (c) Placing laser spot marks is impossible in this case of OCT-guided planning for macular laser photocoagulation. (d) Retinal edema zones (total area, 13.12 mm^2^) are identified on the RM-SLO image (white dashed line represents the zone boundary; asterisk represents the foveal center). (e) RM-SLO image is superimposed onto the baseline image. (f) In RM-SLO-guided planning for macular laser photocoagulation, the number of laser spot marks was 198.

Seventeen out of 39 final OCT retinal thickness maps (43.6%) were found to be inadequate for immediate import into the NAVILAS system with the following overlay on a baseline fundus image. The reasons for this were incorrect automated segmentation (with underestimation of the true retinal thickness due to misidentification of outer retinal layers [21, 22]) in 14/17 eyes, misidentification of foveal center (off-center artifact [21, 22]) due to incompliance with the gaze fixation requirement in 2 cases, and the combination of the first two reasons in 1 case (Figure 1). In all these 14 eyes, after manual correction of retinal boundary segmentation errors in OCT B scans, retinal thickness maps were regenerated and found to be adequate (i.e., having no retinal thickness measurement errors) for OCT-guided planning for MLP. In 2 out of the 3 cases of misidentified foveal center, a scan was slightly off-center, and correction was made through manual repositioning of the foveal center, whereas another case required repeating the scan followed by manual repositioning of the foveal center. Neither corrections nor retakes were required for RM-SLO images, all of which were found to be adequate for RM-SLO-guided planning for MLP. In 2/39 eyes, neither of the two retinal specialists managed to identify the zone of edema with confidence and to perform the treatment planning based on OCT findings only. This was caused by the insignificant edema height (and, consequently, poor identification of the edema on the color map) in one case, and both the out-of-scan-area location of retinal edema and the insignificant edema height in another case (Figure 2). In both these eyes, the retinal edema was clearly visualized on RM-SLO images, which allowed for RM-SLO-guided planning for MLP (Figure 2); these eyes were excluded from statistical analysis. In the rest of the eyes (*n* = 37; 94.9%), rather large intraretinal cysts (i.e., larger than those at the adjacent extrafoveal sites) were found in the foveal zone on RM-SLO images.

### 3.2. Measurement of the Retinal Edema Area

**Figure 3:**
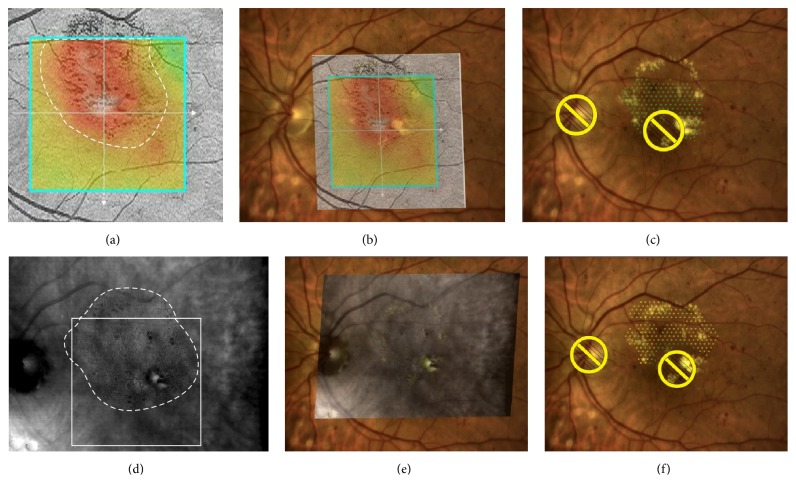
OCT- versus RM-SLO-guided treatment plan for macular laser photocoagulation. (a) OCT retinal thickness map fails to cover the entire zone of retinal edema (area, 9.33 mm^2^). (b) OCT map superimposed onto the baseline image. (c) The number of laser spots placed in OCT-guided treatment planning is 149. (d) The entire zone of retinal edema is identified on RM-SLO image (entire edema area, 14.51 mm^2^; white dashed line represents the edema zone boundary; white square represents a 5 mm × 5 mm zone involved into OCT retinal thickness map; the area of edema identified by RM-SLO within the 5 mm × 5 mm zone is 10.18 mm^2^). (e) RM-SLO image superimposed onto the baseline image. (f) The number of laser spots placed in RM-SLO-guided treatment planning is 219 (i.e., more than that in OCT-guided treatment planning).

The area of edema on RM-SLO image was found to be statistically significantly larger than that on OCT map of the same eye by each physician (physician 1, *P* = 0.004; physician 2, *P* = 0.003). The average area of edema on RM-SLO image was larger than that on OCT map (14.5 ± 4.3 mm^2^ versus 9.1 ± 1.9 mm^2^, *P* = 0.015). However, in 24 eyes (61.5%), OCT mapping did not allow to visualize the entire area of retinal edema, since the edema territory exceeded the boundaries of a 5 mm × 5 mm scan area (Figure 3). Therefore, in these eyes, the comparison of the retinal edema area was redone for OCT retinal thickness map and RM-SLO image solely within a 5 mm × 5 mm scan area aligned against the foveal center (Figure 3).

The retinal edema area measured within a 5 mm × 5 mm zone of RM-SLO image (i.e., within a zone identical to a 5 mm × 5 mm OCT scan area) was not statistically significantly different from that measured on OCT of the same eye by each physician (physician 1, *P* = 0.25; physician 2, *P* = 0.28). The average retinal edema area measured within a 5 mm × 5 mm zone of RM-SLO image was statistically insignificantly larger than that measured on OCT maps (10.5 ± 2.2 mm^2^ versus 9.1 ± 1.9 mm^2^, *P* = 0.11).

In comparison of measuring edema area on RM-SLO images, there was no statistically significant difference among the 2 physicians (*P* = 0.17). No statistically significant difference among the 2 physicians was also present in comparison of measuring edema area on OCT maps (*P* = 0.19).

### 3.3. Spot Placing

The average number of spots using RM-SLO images and OCT maps was 189.6 ± 77.4 and 136.6 ± 46.8, respectively, *P* = 0.003 (Figure 3). In comparison of placing laser spots based on RM-SLO images, there was no statistically significant difference among the two physicians (*P* = 0.13). No statistically significant difference among the 2 physicians was also present in comparison of placing laser spots based on OCT maps (*P* = 0.19).

## 4. Discussion

This study demonstrates that MLP treatment planning for diabetic or RVO-related ME can be guided by RM-SLO, with the spots placed at the zones of visualized intraretinal microcysts, and these zones generally conforming in size and shape to those of increased retinal thickness (secondary to edema) on OCT.

A key RM-SLO feature is immediate visualization of retinal edema, whereas in OCT mapping and FA, the presence of edema is indicated only by phenomena, increased retinal thickness and dye leakage, respectively. When the entire edema zone was within a 5 mm × 5 mm region (thus corresponding to the limitation imposed by OCT), there was no statistically significant difference between the areas of retinal edema visualized by RM-SLO and that found by OCT (however, the average of the former area was somewhat higher than that of the latter).

Previous studies have revealed no advantage of FA-guided over OCT-guided MLP based on comparison of different characteristics including the area of retinal edema identified by the method [16]. Additionally, Vujosevic et al. [19] have confirmed that RM-SLO does not suffer from the disadvantages characteristic for FA. Therefore, it can be expected that RM-SLO-guided planning for MLP will show comparable results to those seen in FA-guided planning. Moreover, the use of RM-SLO images can offer some advantages in planning for navigated MLP compared with the use of OCT retinal thickness maps.

As opposed to the OCT retinal thickness map, the RM-SLO image, allows visualizing the edema within the central fundus, which is seen on the baseline image. A NAVILAS-obtained fundus photograph corresponds to the field of view of 50°, while a standard OCT retinal thickness map covers 18° or 22° (depending on the OCT system, this corresponds to 5 mm × 5 mm or 6 mm × 6 mm ETDRS macular maps), [23] and an RM-SLO image covers up to 60°. No possibility to visualize the entire retinal edema zone on the OCT retinal thickness map was found in 61.5% of the eyes of our study. At the same time, the RM-SLO image allows visualizing the retinal edema within the entire central fundus; consequently, the area of edema measured in RM-SLO-guided treatment planning was found to be statistically significantly larger than that in OCT-guided treatment planning. Moreover, the RM-SLO image includes the optic nerve and the vascular arcades, thus facilitating the overlaying of images.

The average area of retinal edema revealed with RM-SLO within a 5 mm × 5 mm fundus zone was statistically insignificantly larger than that revealed with OCT. This difference may result from the fact that RM-SLO allows visualizing any small intraretinal microcysts located at the boundary of edema zone, with these microcysts either not increasing retinal thickness or increasing it insignificantly. Consequently, in OCT mapping, the retinal thickness in these microcystic regions is shown with a “nearly normal” color, and the regions may receive no spot marking at the time of planning for MLP.

Large cysts (such as those detected with RM-SLO in the foveal center in most of the eyes of our study) may serve as an additional landmark for identification of the foveal center, which has been confirmed by Yamamoto et al. [20]. Therefore, in RM-SLO-guided planning for MLP, the foveal center is identified not only on the baseline color image produced by NAVILAS system, but also with the help of additional landmarks, which allows avoiding its mistaken photocoagulation. At the same time, the foveal center is identified on the OCT retinal thickness map indirectly, using the fixation point.

A fixation error, if any, may cause the displacement of the foveal center marker from the true center and the displacement of the entire retinal thickness map with respect to the background SLO-type fundus photograph. Consequently, both the edema region and the foveal center will be identified incorrectly on the final retinal thickness map with respect to the background SLO-type fundus photograph. This may result in incorrect (e.g., outside-of-edema) placement of spot marks at the time of planning for MLP, since the retinal thickness map of the macula is superimposed onto the baseline color image (produced by NAVILAS system) using the landmarks of the background SLO-type fundus photograph. The requirements for a gaze fixation and no blinking during scan acquisition (acquisition time specified in the EMM5 protocol is 1.3 s) may be another cause for the above mentioned displacement of the foveal center from the true center. At the same time, acquisition of RM-SLO image occurs instantly, and requires neither a gaze fixation nor blinking inhibition, since the image covers most of the fundus area and can be aligned onto the baseline image.

If the edema results in an insignificant increase in retinal thickness (2 eyes of the study), the edema regions are not clearly defined on the OCT retinal thickness map and barely differ in color from non-edematous regions on the color map. Such edema regions become even less noticeable against the background SLO-type fundus photograph which is required for superimposition of the OCT retinal thickness map onto the baseline image.

In a considerable proportion of cases, OCT retinal thickness maps include underestimated areas of retinal thickening, which makes them inadequate for immediate import into the NAVILAS system and planning for MLP. This is associated with the attenuation of the signal by highly reflective intraretinal masses (hard exudates and hemorrhages which are often found in diabetic and proliferative retinopathy patients, potential candidates for MLP), with a correspondingly decreased signal of the retinal pigment epithelium. In such cases, the software algorithm misidentifies these highly reflective intraretinal masses as retinal pigment epithelium, uses them for segmentation of outer retinal layers, and computes underestimated retinal thickness values. Schneider et al. have found such errors in OCT images in 35.3% of examined patients with diabetic retinopathy [24], which is insignificantly less than the percentage found in our study. The difference may result from the inclusion of patients with RVO (because intraretinal hemorrhages are typical for central RVO pathology) and use of other OCT system in our study.

RM-SLO-guided planning for MLP has several disadvantages. First, a lower vessel contrast observed on the RM-SLO image compared to that on the SLO-type image produced by RTVue-100, resulting in an insignificant delay in the image superimposition process. Second, RM-SLO does not allow the physician to make an adequate conclusion about the post-treatment trends in edema-related parameters, since mean foveal thickness and macular volume, the indices providing the most valuable information for quantitative monitoring of ME, can be only measured with OCT. Hence, the use of RM-SLO does not exclude the need for the use of OCT. The correction of the automated segmentation boundaries and foveal center position for a later comparison of the pre-treatment OCT map with those obtained at different post-treatment time points, however, can be performed at any time before the comparison. Third, the measurement of retinal edema area based on RM-SLO images requires a certain level of expertise.

En face OCT imaging combines a number of advantages of both RM-SLO and OCT mapping. This approach allows the physician to visualize retinal microcysts in the macular region and to obtain quantitative data related to edema (e.g., mean foveal thickness) simultaneously. En face OCT, however, suffers all the disadvantages of conventional OCT related to the performance of scan protocol (automated segmentation errors, gaze fixation requirement, and a limited scan area).

The study has a number of limitations. First, we used only one OCT device. Since occurrence of different artifacts varies widely among OCT devices depending also on pathology, one may however expect occurrence of similar difficulties in planning for MLP when guided by the data derived from images taken with other OCT devices [25]. The limitation mentioned is not relevant to RM-SLO, since currently, F10 is the only commercially available scanning laser ophthalmoscope with retro-mode. Second, the study involved a relatively small number of eyes and did not involve controls for the comparison of anatomic and functional outcomes. Therefore, whether a larger photocoagulated area in MLP for ME results in better visual outcomes or a more sustained effect is so far unknown and warrants further investigation in a prospective controlled study.

In conclusion, our study demonstrates (1) the possibility of planning for navigated MLP based on the RM-SLO image overlayed on the fundus photograph of the same eye, (2) larger treatment areas suggested by RM-SLO-guided MLP plans for ME compared to those suggested by OCT-guided plans and (3) narrow variability in treatment planning among the retina specialists.
